# Temporal trends of geographic variation in mortality following cancer diagnosis: a population-based study

**DOI:** 10.1186/s12889-018-6353-1

**Published:** 2019-01-07

**Authors:** Yakir Rottenberg, Aviad Zick, Hagai Levine

**Affiliations:** 1 The Department of Oncology, Hadassah-Hebrew University Medical Center, and Hebrew University-Hadassah Medical School, 91120 Jerusalem, Israel; 2 The Jerusalem Institute of Aging Research, Hadassah-Hebrew University Medical Center, Mount Scopus, and Hebrew University-Hadassah Medical School, 91120 Jerusalem, Israel; 3 Hebrew University-Hadassah Braun School of Public Health and Community Medicine, Ein Kerem, 91120 Jerusalem, Israel

**Keywords:** Health services, Cohort study, Geographic variations, Peripheral regions, Cancer, Inequalities

## Abstract

**Background:**

Inequalities among the western population, combined with the introduction of new treatment options for cancer, have challenged endeavors to provide equal care to patients with cancer. Israel’s highly developed healthcare system and mandatory National Health Insurance afforded an opportunity to study geographic variation over time in mortality following cancer diagnosis.

**Methods:**

This historical prospective cohort study included a nationally representative cohort that was assessed by the Israeli Central Bureau of Statistics 1995 census and followed until 2011. The cancer incidence (1995–2009) was ascertained by the Israel National Cancer Registry. We analyzed the effect on patient outcome of living in a given district, according to the Israeli Central Bureau of Statistics classification. Patients were stratified by the year of diagnosis (1995–1997, 1998–2000, etc.), and associations were adjusted for age, ethnicity, and districts. We excluded patients with malignancies associated with screening program (breast, prostate, colon, and cervical cancers).

**Results:**

This study included 26,173 patients living in 13 residential districts. During the last years (2007–2009) of the study, the hazard ratio (HR) for risk of death was high in 8/13 districts (61.5%), compared to 4/13 (30.7%) during 2004–2006, and 0/13 (0%) during 2001–2003. Districts that were less likely to be associated with increased risk of death were located in the center of Israel and in metropolitan areas, compared to the peripheral regions. Furthermore, HRs were substantially higher in the last years of the study (2007–2009, HRs rose to 1.69, 95%CI: 1.38–2.08) compared to the earlier years (2004–2006, HRs rose to 1.35, 95%CI: 1.13–1.62).

**Conclusion:**

Our findings suggested that geographic variation for mortality following cancer diagnosis have increased over time. Our results provide policy makers with vital information regarding the need for targeted interventions, mainly in peripheral regions.

**Electronic supplementary material:**

The online version of this article (10.1186/s12889-018-6353-1) contains supplementary material, which is available to authorized users.

## Background

Limited access to cancer care services is a significant barrier faced by residents of peripheral, remote regional communities [1]. In peripheral areas, patients with cancer travel long distances to major cancer centers for diagnosis, treatment, and follow-up. Consequently, they incur out-of-pocket costs for traveling and accommodations [2]. In addition, both patients and their families frequently face disruptions in their daily routines. Lack of access to efficient health care services may be one reason for the disparity in survival among various districts [1].

Residence in a non-metropolitan district may serve as a marker for deprived socioeconomic environments. Socioeconomic variables have been found to be associated with cancer survival for several type of cancer. Thus, patients with cancer that live in affluent regions have shown higher survival rates than patients that live in deprived regions [3]. This association was validated, even in countries with rather comprehensive access to health care for all population groups [3–6].

Over the last several years, the relative survival of patients with cancer has, in general, steadily increased over time [7]. At the same time, the introduction of novel anti-cancer regimens has changed the therapeutic options for many patients significantly, but it has also given rise to different profiles of care. For example, tyrosine kinase inhibitors can be given orally on a daily basis at a fixed dose, but this regimen requires continual care by family physicians [8], because the use of these agents has been frequently associated with fatigue [8]. By reducing the patients’ quality of life, side effects often lead to a discontinuation of treatment, which results in suboptimal efficacy [8].

Patients diagnosed with cancer face problems often associated with chronic diseases. These problems include multiple, changing symptoms due to the cyclical nature of cancer, repeated hospital appointments, dealing with uncertainty and the need to integrate various hospital and community services [9]. Indeed, several barriers exist that impede the efficient use of primary care services by cancer patients who takes oral anti-cancer medications. Many patients believe their family physician lacks the expertise to support them, due to the complexity and variability in treating patients diagnosed with cancer [9, 10].

Israel affords an opportunity to assess mortality risks following a cancer diagnosis among various residential districts over time, because it has a highly developed health care system [11], mandatory national health insurance [11], and uniform cancer registration. Indeed, much effort has been directed by the Israeli government to reduce the gaps between the country’s periphery and center in the health sector [12].

Thus, this study investigated the following hypotheses: (1) geographic variations in mortality risk among districts will increase over the study period; and (2) mortality risk following a cancer diagnosis are likely to be elevated in peripheral districts compared to metropolitan areas. The use of large national databases allowed us to adjust the results for potential confounders and to mitigate selection and information biases.

## Methods

### Study population

This study was designed as a historical prospective cohort study. Cohort inception and baseline measurements were acquired from the Israeli Central Bureau of Statistics 1995 census [13]. The study population included a representative sample of the entire population that completed a comprehensive interview (20% of the population in Israel, aged 15 years and over).

### Cancer incidence

Data on the cancer incidence were ascertained with the Israel National Cancer Registry, updated to 2010. The registry was established in 1960, and since 1982, it has received compulsory notifications of cancer incidence, by law. Notifications include data from numerous sources, including pathology reports, discharge summaries, and death certificates. The completeness of the registry was found to be about 95% for solid tumors [13]. All patients diagnosed with cancer between January 1995 and December 2009 were included in the current study. Due to possible variability among various districts in the diagnosis rates of malignancies associated screening program (breast, colorectal, prostate and cervix) [14], these malignancies were excluded from the main analyses to mitigate the risks of lead-time bias and length-time bias. Lung cancer was included in the current study, because the screening program was not available during the study period. Sensitive analyses were carried which included all cancers (without excluding malignancies associated screening program). In addition, patients diagnosed with cancer before 1995 were excluded from the current study.

### Study variables

We assessed variables related to mortality risk after a diagnosis of cancer, including: age, sex, ethnicity (self-reported Jewish vs. non-Jewish), and district. Israel is a small country (only 22,072 km^2^) that includes 14 districts, according to the Israeli Central Bureau of Statistics. For this analysis, we merged two districts into single district, due to the low number of cancer cases during the study period. Thus, this study evaluated a total of 13 districts. Metropolitan areas (Jerusalem, Tal-Aviv, Haifa and Beer-Sheba) were labeled according to the Central Bureau of Statistics definition [15]. The Jerusalem district was considered the reference district according the Israeli Central Bureau of Statistics’s rank. In addition, further adjusted analyses were carried in order to reveal the impact of residential socioeconomic score (ordinal variable, based on the town/city of residence, according to a national classification of 10 clusters by geographical units) on the study’s outcomes.

### Survival outcome

Survival outcome was measured from the date of diagnosis until the date of death or December 31st 2011, whichever came first. In order to evaluate changes in mortality during the study’s period, combined with a small number of cancer cases in some peripheral districts, we stratified results into 3 years group. Mortality was determined from data in the Israel Population Registry, Central Bureau of Statistics - Cause of Death File, updated to the end of 2011. Mortality data are considered to be 100% complete for all individuals that died in Israel. In addition, sensitive analyses were carried which assessed cancer mortality (rather than all-cause mortality).

### Statistical analyses

We compared cancer survival by and age-adjusted mortality rates and by constructing multivariate models. We controlled for age, sex, ethnicity, and district with a Cox Proportional hazards analysis. We verified the proportional hazards assumption by inspecting log-minus-log plots.

The year of diagnosis was stratified into groups of 3 years (i.e., 1995–1997, 1998–2000, etc.). The mortality hazards ratio (HR) was calculated with reference to the reference district (Jerusalem district). For all analyses, *p* < 0.05 was considered statistically significant. The SPSS program (18th version; Chicago, Illinois) was used for all statistical analyses.

## Results

The study population included 26,173 patients diagnosed with cancer not associated screening program during the years 1995–2009. Baseline characteristics of the study population are described in Table 1. The study population included mostly males (*n* = 13,749, 52.2%), and most individuals were of Jewish ethnicity (*n* = 23,701, 90.6%). Lung cancer was the most frequent malignancy (12.2%), followed by melanoma (10.6%), bladder cancer (9.4%), and leukemia (8.8%). Confirmed data on staging at diagnosis were not available for about one-third of the patients (*n* = 9659, 36.9%). Only a minority of patients (*n* = 3780, 14.4%) were diagnosed with confirmed metastatic disease.

**Table 1:** Characteristics of the study population (*n* = 26,173)

Study variable	Value
*Demographic variables*	
Age, years [mean (±SD)]	64.9 ± 15.6
Male, %	52.5%
Jew, %	90.6%
*Cancer staging at diagnosis*	
Metastasis at diagnosis, %	14.4%
Unknown stage at diagnosis, %	36.9%
*Common Cancer sites*	
Lung cancer, %	12.2%
Melanoma, %	10.6%
Bladder cancer, %	9.4%
Leukemia, %	8.8%
Lymphoma, %	6.6%
Brain malignancies, %	6.3%
Gastric cancer, %	5.6%
Pancreas cancer, %	4.7%
Renal cancer, %	4.7%
Endometrial cancer, %	4.3%
Ovarian cancer, %	3.2%

During the study period, 15,116 patients died (57.8%). Improvement in survival following cancer diagnosis was seen throughout the study’s period (Additional file 1: Table S1). Death following cancer diagnosis (Table 2) was associated with age (increased risk), sex (higher risk in men), and ethnicity (lower risk among Jews). Most districts associated with increased risk of death were in the north (4/5 districts) and the south (1/1 district), in contrast to districts in the center of Israel and in metropolitan areas (1/4 and 1/3 districts, respectively). Similar trend was seen also in the age-adjusted mortality rates (Additional file 1: Table S2).

**Table 2:** Adjusted^a^ all- cause mortality hazards ratios, stratified to year of diagnosis

Variables	All	1995–1997	1998–2000	2001–2003	2004–2006	2007–2009
N	26,173	4466	5063	5313	5596	5735
Age (per year)	1.052 (1.050–1.053) ^***^	1.055 (1.052–1.058) ^***^	1.050 (1.047–1.052) ^***^	1.051 (1.048–1.054) ^***^	1.051 (1.048–1.054) ^***^	1.054 (1.050–1.057)^***^
Sex (male)	1.11 (1.08–1.15) ^***^	1.16 (1.08–1.24) ^***^	1.16 (1.08–1.240) ^***^	1.16 (1.08–1.250^***^	1.05 (0.98–1.13)	1.02 (0.95–1.11)
Ethnicity (Jew)	0.72 (0.68–0.77) ^***^	0.67 (0.58–0.77) ^***^	0.79 (0.69–0.90) ^**^	0.70 (0.61–0.79) ^***^	0.72 (0.63–0.82) ^***^	0.69 (0.60–0.79) ^***^
Districts	13 df^b^ (*p* < 0.001)	13 df^b^ (*p* = 0.121)	13 df^b^ (*p* = 0.010)	13 df^b^ (*p* = 0.097)	13 df^b^ (*p* = 0.003)	13 df^b^ (*p* < 0.001)
*Metropolises*						
Jerusalem	1	1	1	1	1	1
Tel Aviv	1.03 (0.96–1.09)	1.02 (0.88–1.18)	0.98 (0.86–1.13)	0.88 (0.77–1.01)	1.01 (0.90–1.16)	1.28 (1.09–1.50) ^**^
Haifa	1.00 (0.94–1.08)	0.94 (0.80–1.01)	0.95 (0.82–1.11)	0.91 (0.78–1.06)	1.09 (0.94–1.27)	1.12 (0.94–1.33)
BeerSheva	1.25 (1.14–1.36) ^***^	1.21 (0.99–1.74)	1.22 (1.02–1.47) ^*^	0.97 (0.80–1.18)	1.24 (1.03–1.51) ^*^	1.69 (1.38–2.08) ^***^
*Center*						
Rehovot	1.00 (0.92–1.09)	0.97 (0.80–1.17)	1.05 (0.88–1.27)	0.91 (0.76–1.10)	1.01 (0.84–1.22)	1.06 (0.96–1.31)
Hasharon	1.05 (0.95–1.15)	1.01 (0.89–1.35)	0.98 (0.80–1.20)	0.83 (0.79–1.02)	1.32 (1.08–1.62) ^**^	1.09 (0.86–1.38)
Petach Tikva	1.01 (0.93–1.09)	0.99 (0.83–1.18)	0.98 (0.83–1.17)	0.89 (0.75–1.06)	0.98 (0.82–1.18)	1.23 (1.01–1.50) ^*^
Ramla	1.25 (1.11–1.40) ^***^	1.15 (0.89–1.49)	1.07 (0.83–1.39)	1.17 (0.93–1.47)	1.26 (0.98–1.61)	1.67 (1.26–2.20) ^***^
*South*						
Ashkelon	1.24 (1.14–1.35) ^***^	1.22 (1.02–1.47) ^*^	1.20 (1.01–1.77) ^*^	1.01 (0.91–1.31)	1.35 (1.13–1.62) ^***^	1.31 (1.06–1.61) ^*^
*North*						
Hadera	1.17 (1.05–1.29) ^**^	1.13 (0.89–1.44)	1.15 (0.91–1.45)	0.97 (0.77–1.21)	1.16 (0.92–1.47)	1.48 (1.15–1.89) ^**^
Akko	1.15 (1.05–1.25) ^**^	1.05 (0.85–1.29)	1.26 (1.04–1.52) ^*^	1.00 (0.83–1.22)	1.22 (1.00–1.48) ^*^	1.22 (0.98–1.51)
Izrael	1.15 (1.04–1.27^**^	1.12 (0.90–1.40)	1.07 (0.86–1.34)	1.05 (0.85–1.30)	1.16 (0.93–1.45)	1.34 (1.06–1.70) ^*^
Tzfat	1.04 (0.68–1.24)	0.94 (0.60–1.45)	0.92 (0.61–1.37)	0.79 (0.53–1.18)	1.12 (0.75–1.67)	1.58 (1.07–2.34) ^*^
Kineret	1.22 (1.02–1.45) ^*^	1.01 (0.70–1.75)	1.01 (0.94–1.81)	1.05 (0.69–1.61)	1.12 (0.76–1.65)	1.45 (0.99–2.12)

^*^p < 0.05 ^**^p < 0.01 ^***^p < 0.0001

^a^Adjusted for age, sex, ethnicity and districts

^b^ degree of freedom

**Fig. 1:**
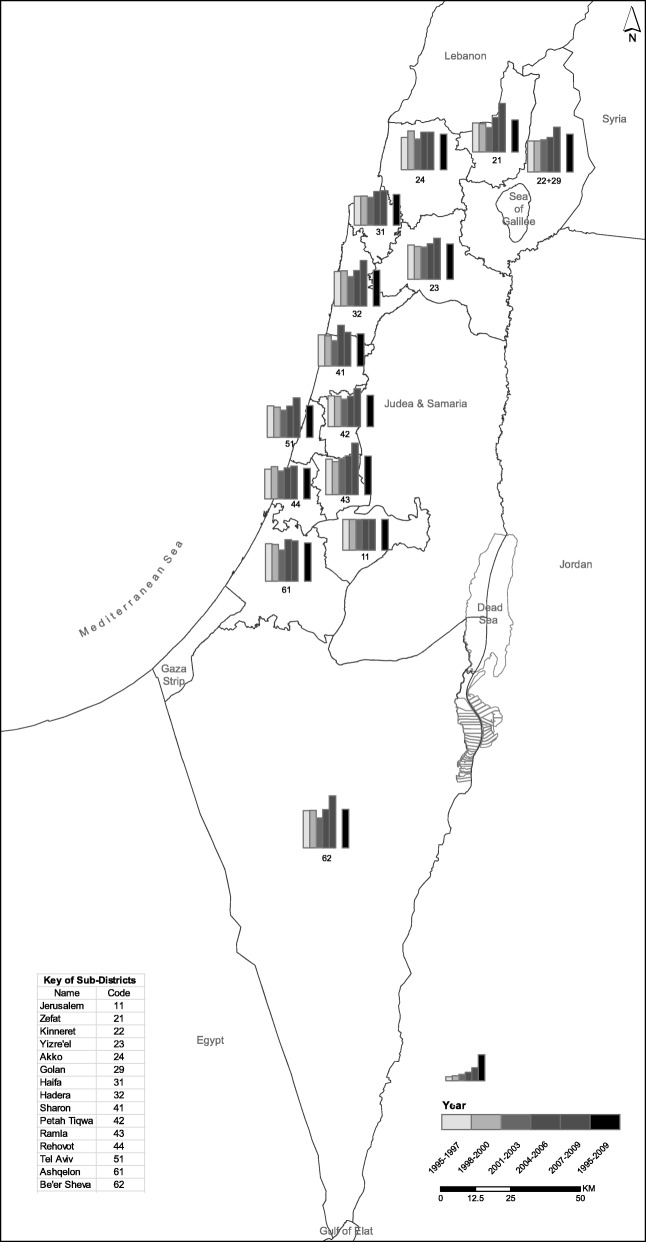
Hazard ratio for death according to districts adjusted for age, sex and ethnicity, stratified to years of diagnosis (reference group: Jerusalem = 1.00)

Stratification of the year of diagnosis (Fig. 1 and Table 2) revealed that, between the 1998–2000 period and the 2001–2003 period, the districts showed diminished differences in the risk of death following cancer diagnosis. Indeed, in the years 2001–2003, no district was associated with an increased risk of death (*p* = 0.097, 13df). In contrast, increased risks were observed during 2004–2006 in 4/13 districts (*p* = 0.003, 13df). This trend was accentuated during the last years of the study (2007–2009), when increased risks of death were observed in 8/13 districts (*p* < 0.001, 13df). Furthermore, the magnitude of the effect was substantially higher in the last years of the study compared to prior years. For example, the maximum HR of death among patients diagnosed with cancer was 1.69 (95%CI: 1.38–2.08) for the 2007–2009 period, as opposed to 1.35 (95%CI: 1.13–1.62) for the 2004–2006 period. In the analyses including residential socioeconomic score, increases differences among districts for death following cancer diagnosis was also seen mainly in the last years (2007–2009 period) of the study’s period (Additional file 1: Table S3). At this period, the significant of the association between residential socioeconomic score and mortality was stronger. In addition, during the 2007–2009 period differences among districts for death following cancer diagnosis was seen also among metropolitan areas. Analyses restricted to cancer death (Table 3) revealed similar trends in the lasts years of the study; during the years 2001–2003 no district was associated with increased risk of cancer death, while during the 2004–2006 elevated risks were observed in 5/13 districts and in 9/13 districts during the years 2007–2009. On the other hand, this trend was attenuated in the analyses which included also cancers associated with screening program (Additional file 1: Table S4). For example, similar *p values* (*p* = 0.001, 13df) were seen during all the study’s period, except to years 2007–2009 (*p* < 0.001, 13df). In addition, interaction analyses (Additional file 1: Tables S5 and S6) included lots of cells (including the interaction and the districts), and seems that these interactions added limited information to the models due to decreased power.

**Table 3:** Adjusted^a^ cancer mortality hazards ratios, stratified to year of diagnosis, excluding screening associated cancers

Variables	All	1995–1997	1998–2000	2001–2003	2004–2006	2007–2009
N	26,173	4466	5063	5313	5.596	5735
Age (per year)	1.042 (1.041–1.044)^***^	1.044 (1.041–1.047)^***^	1.039 (1.036–1.042)^***^	1.038 (1.035–1.041)^***^	1.043 (1.040–1.047)^***^	1.050 (1.046–1.053)^***^
Sex (male)	1.09 (1.05–1.13)^***^	1.13 (1.04–1.23)^**^	1.13 (1.05–1.23)^**^	1.15 (1.06–1.24)^**^	1.01 (0.93–1.10)	1.01 (0.92–1.10)
Ethnicity (Jew)	0.71 (0.66–0.76)^***^	0.66 (0.56–0.77)^**^	0.84 (0.63–0.86)^***^	0.71 (0.62–0.83)^***^	0.73 (0.63–0.84)^***^	0.66 (0.56–0.77)^***^
Districts	13 df^b^ (*p* < 0.001)	13 df^b^ (*p* = 0.43)	13 df^b^ (*p* = 0.04)	13 df^b^ (*p* = 0.01)	13 df^b^ (*p* = 0.04)	13 df^b^ (*p* < 0.001)
*Metropolises*						
Jerusalem	1	1	1	1	1	1
Tel Aviv	1.06 (0.98–1.14)	0.96 (0.81–1.13)	0.97 (0.83–1.14)	0.89 (0.76–1.05)	1.08 (0.93–1.27)	1.44 (1.19–1.73)^***^
Haifa	1.00 (0.93–1.09)	0.90 (0.75–1.08)	0.96 (0.81–1.15)	0.88 (0.74–1.05)	1.10 (0.93–1.31)	1.16 (0.94–1.43)
BeerSheva	1.31 (1.18–1.44)^***^	1.10 (0.88–1.39)	1.31 (1.06–1.62)^*^	1.03 (0.83–1.28)	1.38 (1.11–1.72)^**^	1.83 (1.44–2.32)^***^
*Center*						
Rehovot	1.06 (0.98–1.14)	0.91 (0.73–1.14)	0.97 (0.78–1.19)	0.84 (0.68–1.05)	1.02 (0.82–1.27)	1.28 (1.01–1.62)^*^
Hasharon	1.04 (0.93–1.16)	1.07 (0.84–1.35)	0.98 (0.77–1.25)	0.76 (0.59–0.96)^*^	1.29 (1.01–1.63)^*^	1.21 (0.92–1.59)
Petach Tikva	1.23 (1.08–1.40)^**^	0.99 (0.81–1.21)	1.00 (0.82–1.22)	0.87 (0.71–1.06)	1.06 (0.86–1.30)	1.33 (1.05–1.68)^**^
Ramla	0.99 (0.90–1.10)	1.01 (0.82–1.48)	0.98 (0.72–1.34)	1.20 (0.92–1.56)	1.22 (0.93–1.64)	1.37 (1.20–2.32)^**^
*South*						
Ashkelon	1.27 (1.15–1.40)^***^	1.15 (0.93–1.42)	1.21 (0.99–1.49	1.14 (0.92–1.41)	1.50 (1.22–1.84)^***^	1.31 (1.03–1.68)^*^
*North*						
Hadera	1.18 (1.05–1.33)^**^	1.07 (0.82–1.41)	1.07 (0.81–1.41)	0.93 (0.72–1.21)	1.27 (0.97–1.65)	1.71 (1.29–2.26)^***^
Akko	1.13 (1.02–1.26)^*^	1.00 (0.79–1.27)	1.20 (0.96–1.50)	0.99 (0.79–1.24)	1.26 (1.00–1.58)^*^	1.25 (0.97–1.61)
Izrael	1.21 (1.08–1.36)^**^	1.13 (0.88–1.45)	1.11 (0.86–1.43)	1.11 (0.87–1.41)	1.31 (1.02–1.68)^*^	1.44 (1.10–1.89)^**^
Tzfat	1.02 (0.82–1.26)	0.87 (0.52–1.44)	0.84 (0.52–1.36)	0.66 (0.40–1.09)	1.17 (0.74–1.86)	1.84 (1.18–2.86)^**^
Kineret	1.22 (1.00–1.49)^*^	0.86 (0.48–1.54)	1.22 (0.83–1.80)	1.12 (0.71–1.80)	1.39 (0.92–2.09)	1.74 (0.95–2.29)

^*^p<0.05 ^**^p<0.01 ^***^p<0.0001

^a^Adjusted for age, sex, ethnicity and districts

^b^degree of freedom

## Discussion

Our results partially confirmed the hypotheses investigated in this study. We found that increases in geographic variation for all-cause mortality and cancer death following cancer diagnosis, mainly during the last years of the study. In addition, only a minority of districts in the center of Israel and the metropolitan areas (1/4 and 1/3, respectively) were associated with increased risk of all-cause mortality, in contrast to the majorities of districts in the north (4/5) and south (1/1). Nevertheless, the risk of mortality was attenuated in various districts between the 1998–2000 and 2001–2003 periods.

The mitigated geographic variation for all-cause mortality following cancer diagnosis among districts up to 2003 may be explained by the introduction of National Health Insurance [11, 16]. Since 1995, Israel has a National Health Insurance Law, which results in improved health system beyond universal health coverage [11]. Citizens choose from a few competing non-profit health plans, which provide a broad package of benefits stipulated by the government [11]. Indeed, the Israeli health care system has become quite efficient; despite spending a relatively low proportion of the gross domestic product on health care (less than 8%), the country’s health variables are comparable to those of other developed countries [11].

The increased geographic variation of mortality risk observed since 2004 may be explained by several factors. Improvement in survival following cancer diagnosis, together with the introduction of novel therapies [7], have challenged the health system. These advances require repeated visits to cancer centers and efficient integration of hospital, community, and professional primary care services. In addition, the remarkable strides in cancer treatment, which have yielded improvements in patient outcomes, have generated increasing costs [17]. Despite health basket in Israel regarding cancer treatments is one of the richest and best globally, the financial burden of cancer treatment is beyond the price of specific medication. Indeed, it includes advanced molecular analyses, imaging technique, consultation with multi-disciplinary teams and delaying in approval of novel medications of a numerous months by the health basket’s committee. These expenditures may not be covered by the health basket. Consequently, the financial burden has been shifted to patients, which has resulted in higher out-of-pocket expenses [17]. Actually, higher residential socioeconomic score was associated with decreased risk for death following cancer diagnosis in the current study (Additional file 1: Table S3). Indeed, developing financial difficulties during cancer illness has been associated with an increased risk of death in Italy [18]. This dismal outcome was reported although that most of the clinical pathway of cancer patients is covered by the Italian public health system, including inpatient and outpatient services and drugs [18]. In parallel, over the study period, reliance on private financing has grown, with potentially deleterious effects; the proportion of private financing that contributes to total health expenditure has sharply increased from 32% in 1995 to 39% in 2012 [11, 19, 20]; this change may have played a dominant role in the growing geographic variation among the study population.

Our current results may also be explained by increased geographic variation of mortality risk unrelated to cancer diagnosis during the lasts years of the study. However, this hypothesis is not supported by the similar trend which was seen in cancer mortality (Table 3). In addition, a disproportionally high incidence of highly aggressive malignancies during the last years of the study, in some districts may explained the study’s results. Indeed, heterogeneity in several variables among districts may result in changes in the incidence of lung cancer and other aggressive, smoking-related malignancies over the study period. Smoking cessation was associated with multiple variables, include age, marital status [21], ethnicity, and education levels [21, 22]. Consequently, taking into account the long delay between smoking and a lung cancer diagnosis, the changes we observed may have reflected changes that took place during the twentieth century.

Increased risk of mortality following a cancer diagnosis was mainly observed among non- metropolitan districts and districts located outside the center of Israel. Israel is a small country; it is approximately 470 km long, and 135 km at its widest point. The districts located in the central region extend approximately 80 km in length. The current results were consistent with previous publications [23, 24], which highlighted the worst health outcomes among cancer patients that lived in non-metropolitan regions. For example, among patients diagnosed with glioblastoma multiforme, those living in rural zones had larger tumor sizes at diagnosis, lower rates of radiotherapy, and worse survival, compared to patients living in urban zones, even after controlling for potential confounders [22]. Similarly, the present study emphasized the poor outcomes of patients in peripheral districts. Furthermore, these poor outcomes were seen not only among non-metropolitan districts but also in the peripheral metropolitan (BeerSheva district). Consequently, these dismal outcomes which were reported in previous studies were validated in a relatively small country with highly appreciated health services [11, 16], including National Health Insurance coverage [11, 16].

The current study had several strengths. The high-quality dataset and linkage to highly validated databases (Israel Cancer Registry and the Cause of Death File) supported the internal validity of the study. The population-based inception cohort supported the study’s external validity. Furthermore, our exclusion of malignancies associated with screening program (breast, colorectal, prostate, and cervical cancers) reduced the risks of a lead-time bias and a length-time bias. Similar results were seen also in the analyses which assessed cancer mortality, as opposed to analyses which include also malignancies associated with screening program. These findings suggest that the present study assesses the impact of geographic variations on the care of cancer patients rather than the geographic variation of cancer incidence and the implantation of screening programs.

Our study also had some limitations. Because information on staging was only partially available, it was not included in the current analyses. Consequently, we could not assess whether the distribution of late diagnoses among the districts might have explained the current results. In addition, we lacked information on suggested treatments and compliance. Thus, some uncertainty in our results might be due to disparities in treatment options and compliance among the districts. In addition, residual confounding may also have influenced our findings. For example, data on competing comorbidities and functional status were lacking. However, these limitations did not impair the validity of the study results. Lastly, the current study emphasizes geographic variation in mortality following cancer diagnosis, rather than cancer risk and compliance to screening programs which may have greatest impact on cancer morbidity and mortality. Taking into account the high prevalence of cancer, our results may provide important information for those caring for cancer patients and planning health services.

## Conclusion

In conclusion, in this small country, we found increased geographic variations in mortality following cancer diagnosis mainly among various peripheral districts, primarily in the most recent years of this 15-year study. Understanding the complex mechanisms underlying the influence of residential district on the risk of death following a cancer diagnosis remains an important research priority. These results provide important information for planning health and social services. In addition, our findings have clinical ramifications; they suggested that current disease management should be tailored and patient-centered, particularly for patients living in peripheral districts.

## Additional file

Additional file 1: **Table S1.** Adjusted^≠^ all- cause mortality hazards ratios. **Table S2.** Age adjusted mortality rates, stratified to year of diagnosis. **Table S3.** Adjusted^≠^ all- cause mortality hazards ratios, stratified to year of diagnosis. **Table S4.** Adjusted^≠^ all- cause mortality hazards ratios, stratified to year of diagnosis, including screening associated cancers. **Table S5.** Adjusted^≠^ all-cause mortality hazards ratios. **Table S6.** Adjusted^≠^ all-cause mortality hazards ratios. (DOCX 27 kb)

## Abbreviation

HR: Hazards ratio
