# Supplementary material for: Temporal trends of geographic variation in mortality following cancer diagnosis: a population-based study
Source: BMC Public Health. 2019 Jan 7;19:22. doi: 10.1186/s12889-018-6353-1 (PMC6322286; doi:10.1186/s12889-018-6353-1)
Supplement: Supplementary file 1 — Table S1. Adjusted≠ all- cause mortality hazards ratios. Table S2. Age adjusted mortality rates, stratified to year of diagnosis. Table S3. Adjusted≠ all- cause mortality hazards ratios, stratified to year of diagnosis. Table S4. Adjusted≠ all- cause mortality hazards ratios, stratified to year of diagnosis, including screening associated cancers. Table S5. Adjusted≠ all-cause mortality hazards ratios. Table S6. Adjusted≠ all-cause mortality hazards ratios. (DOCX 27 kb) [file 12889_2018_6353_MOESM1_ESM.docx]

| **Table S1: Adjusted^≠^ all- cause mortality hazards ratios** | | | | | | | |
| --- | --- | --- | --- | --- | --- | --- | --- |
| **Study variable** | | | | **HR (95%CI)** | | | |
|  | | | |  | | | |
| **Demographic variables** | | | |  | | | |
| **Age, years [mean (±SD)]** | | | | 1.051 (1.050-1.053) | | | |
| **Male, %** | | | | 1.11 (1.08-1.15) | | | |
| **Jew, %** | | | | 0.69 (0.65-0.73) | | | |
|  | | | |  | | | |
| **Diagnosis year** | | | |  | | | |
| **1995-1997 (reference)** | | | | 1 | | | |
| **1998-2000** | | | | 1.03 (0.98-1.08) | | | |
| **2001-2003** | | | | 0.92 (0.88-0.97) | | | |
| **2004-2006** | | | | 0.91 (0.86-0.95) | | | |
| **2007-2009** | | | | 0.87 (0.83-0.92) | | | |
| ^≠^Adjusted for age, sex, ethnicity and districts. | | | |  | | | |
|  | | | |  | | | |
| **Table S2: Age adjusted mortality rates, stratified to year of diagnosis.** | | | | | | | |
| **Variables** | **1995-1997** | **1998-2000** | **2001-2003** | | **2004-2006** | **2007-2009** |  |
| **N** | 4,466 | 5,063 | 5,313 | | 5,596 | 5,735 |  |
| ***Metropolises*** |  |  |  | |  |  |  |
| **Jerusalem** | 0.697 | 0.626 | 0.598 | | 0.504 | 0.363 |  |
| **Tel Aviv** | 0.677 | 0.627 | 0.539 | | 0.483 | 0.412 |  |
| **Haifa** | 0.660 | 0.629 | 0.564 | | 0.500 | 0.387 |  |
| **BeerSheva** | 0.714 | 0.673 | 0.556 | | 0.508 | 0.471 |  |
| ***Center*** |  |  |  | |  |  |  |
| **Rehovot** | 0.661 | 0.627 | 0.550 | | 0.469 | 0.358 |  |
| **Hasharon** | 0.700 | 0.646 | 0.579 | | 0.590 | 0.375 |  |
| **Petach Tikva** | 0.648 | 0.632 | 0.551 | | 0.469 | 0.400 |  |
| **Ramla** | 0.722 | 0.653 | 0.613 | | 0.526 | 0.517 |  |
| ***South*** |  |  |  | |  |  |  |
| **Ashkelon** | 0.749 | 0.695 | 0.602 | | 0.559 | 0.435 |  |
| ***North*** |  |  |  | |  |  |  |
| **Hadera** | 0.748 | 0.706 | 0.615 | | 0.529 | 0.481 |  |
| **Akko** | 0.704 | 0.701 | 0.628 | | 0.547 | 0.438 |  |
| **Izrael** | 0.715 | 0.671 | 0.645 | | 0.531 | 0.481 |  |
| **Tzfat** | 0.725 | 0.635 | 0.584 | | 0.563 | 0.495 |  |
| **Kineret** | 0.704 | 0.723 | 0.605 | | 0.472 | 0.539 |  |

|  | | | | | | | |
| --- | --- | --- | --- | --- | --- | --- | --- |
| **Table S3: Adjusted^≠^ all- cause mortality hazards ratios, stratified to year of diagnosis.** | | | | | | |  |
| **Variables** | **All** | **1995-1997** | **1998-2000** | **2001-2003** | **2004-2006** | **2007-2009** |  |
| **N** | 26,173 | 4,466 | 5,063 | 5,313 | 5,596 | 5,735 |  |
|  |  |  |  |  |  |  |  |
| **Age (per year)** | 1.052*** | 1.055*** | 1.050*** | 1.051*** | 1.051*** | 1.051*** |  |
| **Sex (male)** | 1.11*** | 1.16*** | 1.16*** | 1.16*** | 1.05 | 1.03 |  |
| **Ethnicity (Jew)** | 1.76*** | 0.70*** | 0.82** | 0.75*** | 0.76*** | 0.74*** |  |
| **Residential socio-economic position ^€^** | 0.96*** | 0.97* | 0.97* | 0.96** | 0.96** | 0.95*** |  |
| **Districts** | 13 df^±^  (p<0.001) | 13 df^±^  p=0.7 | 13 df^±^  p=0.3 | 13 df^±^  p=0.9 | 13 df^±^  p=0.3 | 13 df^±^  (p<0.001) |  |
| ***Metropolises*** |  |  |  |  |  |  |  |
| **Jerusalem** | 1 | 1 | 1 | 1 | 1 | 1 |  |
| **Tel Aviv** | 1.19*** | 1.09 | 1.05 | 0.98 | 1.11 | 1.45*** |  |
| **Haifa** | 1.17** | 0.98 | 0.99 | 0.97 | 1.16 | 1.20* |  |
| **BeerSheva** | 1.23*** | 1.19 | 1.21* | 0.96 | 1.23* | 1.65*** |  |
| ***Center*** |  |  |  |  |  |  |  |
| **Rehovot** | 1.13** | 1.03 | 1.11 | 1.00 | 1.11 | 1.19 |  |
| **Hasharon** | 1.11* | 1.11 | 1.00 | 0.86 | 1.37** | 1.12 |  |
| **Petach Tikva** | 1.20** | 1.07 | 1.05 | 1.00 | 1.08 | 1.40** |  |
| **Ramla** | 1.09 | 1.11 | 1.05 | 1.12 | 1.20 | 1.59** |  |
| ***South*** |  |  |  |  |  |  |  |
| **Ashkelon** | 1.19*** | 1.18 | 1.17 | 1.04 | 1.29** | 1.24* |  |
| ***North*** |  |  |  |  |  |  |  |
| **Hadera** | 1.08 | 1.14 | 1.16 | 0.98 | 1.17 | 1.49** |  |
| **Akko** | 1.06 | 1.03 | 1.24 | 0.99 | 1.19 | 1.18 |  |
| **Izrael** | 1.12* | 1.10 | 1.05 | 1.03 | 1.13 | 1.28* |  |
| **Tzfat** | 1.01 | 0.91 | 0.90 | 0.77 | 1.08 | 1.53* |  |
| **Kineret** | 1.19 | 1.09 | 1.29 | 1.02 | 1.09 | 1.38 |  |
| ^≠^Adjusted for age, sex, ethnicity and districts.  *p<0.05 **p<0.01 ***p<0.0001  ^±^ degree of freedom  ^€^ based on the town/city of residence, according to a national classification of 10 clusters by geographical units | | | | | | |  |

| **Table S4: Adjusted^≠^ all- cause mortality hazards ratios, stratified to year of diagnosis, including screening associated cancers.** | | | | | | |
| --- | --- | --- | --- | --- | --- | --- |
| **Variables** | **All** | **1995-1997** | **1998-2000** | **2001-2003** | **2004-2006** | **2007-2009** |
| **N** | 44,878 | 7,673 | 8,680 | 9,028 | 9,501 | 9,996 |
|  |  |  |  |  |  |  |
| **Age (per year)** | 1.055 (1.054-1.056)*** | 1.055 (1.052-1.057)*** | 1.053 (1.051-1.056)*** | 1.053 (1.051-1.056)*** | 1.054 (1.051-1.057)*** | 1.060 (1.057-1.063)*** |
| **Sex (male)** | 1.12 (1.09-1.15)*** | 1.16 (1.01-1.23)*** | 1.24 (1.17-1.31)*** | 1.13 (1.07-1.12)*** | 1.04 (0.98-1.11) | 1.03 (0.96-1.10) |
| **Ethnicity (Jew )** | 0.71 (0.68-0.75)*** | 0.70 (0.62-0.78)*** | 0.74 (0.66-0.83)*** | 0.67 (0.60-0.75)*** | 0.70 (0.93-0.79)*** | 0.71 (0.62-0.80)*** |
| **Districts** | 13 df^±^  (p<0.001) | 13 df^±^  (p=0.001) | 13 df^±^  (p=0.001) | 13 df^±^  (p=0.001) | 13 df^±^  (p=0.001) | 13 df^±^  (p<0.001) |
| ***Metropolises*** |  |  |  |  |  |  |
| **Jerusalem** | 1 | 1 | 1 | 1 | 1 | 1 |
| **Tel Aviv** | 1.02 (0.97-1.08) | 0.95 (0.85-1.06) | 1.00 (0.89-1.11) | 0.96 (0.85-1.08) | 1.02 (0.90-1.14) | 1.21 (1.05-1.38)** |
| **Haifa** | 1.03 (0.97-1.09) | 0.94 (0.83-1.06) | 1.00 (0.89-1.14) | 1.00 (0.88-1.14) | 1.11 (0.98-1.26) | 1.01 (0.94-1.28) |
| **BeerSheva** | 1.21 (1.12-1.30)*** | 1.17 (1.00-1.37) | 1.12 (1.03-1.34)* | 1.06 (0.90-1.25) | 1.18 (1.00-1.39)* | 1.53 (1.27-1.83)*** |
| ***Center*** |  |  |  |  |  |  |
| **Rehovot** | 1.01 (0.95-1..09) | 1.04 (0.90-1.21) | 1.03 (0.89-1.12) | 0.98 (0.84-1.14) | 0.95 (0.81-1.11) | 1.06 (0.88-1.27) |
| **Hasharon** | 1.06 (0.98-1.15) | 0.95 (0.81-1.12) | 1.09 (0.93-1.29) | 0.97 (0.81-0.16) | 1.13 (0.95-1.35) | 1.16 (0.95-1.42) |
| **Petach Tikva** | 1.04 (0.97-1.11) | 0.97 (0.84-1.12) | 1.08 (0.94-1.24) | 1.03 (0.90-1.19) | 1.00 (0.86-1.17) | 1.09 (0.92-1.30) |
| **Ramla** | 1.31 (1.12-1.45)*** | 1.25 (1.02-1.53)* | 1.12 (0.97-1.48) | 1.29 (1.06-1.57)* | 1.39 (1.12-1.73)** | 1.44 (1.12-1.84)** |
| ***South*** |  |  |  |  |  |  |
| **Ashkelon** | 1.24 (1.16-1.33)*** | 1.26 (1.08-1.46)** | 1.20 (1.04-1.34)* | 1.25 (1.08-1.46)** | 1.24 (1.06-1.45)** | 1.24 (1.03-1.49)* |
| ***North*** |  |  |  |  |  |  |
| **Hadera** | 1.21 (1.11-1.32)*** | 1.17 (0.97-1.42) | 1.32 (1.09-1.58) ** | 1.10 (0.91-1.32) | 1.20 (0.99-1.47) | 1.26 (1.02-1.59)* |
| **Akko** | 1.16 (1.08-1.26)*** | 1.04 (0.88-1.22) | 1.17 (1.00-1.37) | 1.18 (0.99-1.39) | 1.24 (1.05-1.47)* | 1.19 (0.98-1.44) |
| **Izrael** | 1.22 (1.13-1.33)*** | 1.22 (1.02-1.45)* | 1.13 (0.94-1.34) | 1.17 (0.98-1.39) | 1.15 (0.96-1.38) | 1.54 (1.26-1.88)*** |
| **Tzfat** | 1.08 (0.93-1.25) | 1.00 (0.72-1.38) | 1.21 (0.89-1.66) | 0.92 (0.67-1.27) | 1.00 (0.71-1.42) | 1.34 (0.93-1.94) |
| **Kineret** | 1.30 (1.13-1.51)*** | 1.22 (0.86-1.74) | 1.38 (1.04-1.82)* | 1.12 (0.83-1.73) | 1.26 (0.93-1.72) | 1.45 (1.04-2.03)* |
| ^≠^Adjusted for age, sex, ethnicity and districts.  *p<0.05 **p<0.01 ***p<0.0001  ^±^ degree of freedom | | | | | | |

**Table S5: Adjusted^≠^ all-cause mortality hazards ratios**

| **Study variable** | **HR** | **(95% CI)** |
| --- | --- | --- |
|  |  |  |
| **Demographic variables** |  |  |
| Age, years [mean (±SD)] | 1.051 | (1.050-1.053) |
| Male, % | 1.11 | (1.08-1.15) |
| Jew, % | 0.69 | (0.65-0.73) |
|  |  |  |
| **Diagnosis years** |  |  |
| 1995-1997 (reference) | 1 |  |
| 1998-2000 | 1.03 | (0.98-1.08) |
| 2001-2003 | 0.92 | (0.88-0.97) |
| 2004-2006 | 0.91 | (0.86-0.95) |
| 2007-2009 | 0.87 | (0.83-0.92) |
|  |  |  |
| **Districts** |  |  |
| Jerusalem (reference) | 1 |  |
| Tzfat | 0.93 | (0.60-1.44) |
| Kineret | 1.1 | (0.69-1.76) |
| Izrael | 1.12 | (0.90-1.40) |
| Akko | 1.05 | (0.85-1.29) |
| Haifa | 0.94 | (0.80-1.10) |
| Hadera | 1.13 | (0.89-1.44) |
| Hasharon | 1.08 | (0.88-1.34) |
| Petach-Tikvah | 0.98 | (0.82-1.17) |
| Ramla | 1.14 | (0.88-1.47) |
| Rehovot | 0.96 | (0.80-1.16) |
| Tel-Aviv | 1.01 | (0.88-1.17) |
| Ashkelon | 1.21* | (1.00-1.45) |
| Beer-Sheva | 1.19 | (0.98-1.45) |
| **Districts * year** |  |  |
| Jerusalem*1998-2000 (reference) | 1 |  |
| Tzfat*1998-2000 | 1.01 | (0.56-1.83) |
| Kineret*1998-2000 | 1.19 | (0.68-2.10) |
| Izrael*1998-2000 | 0.94 | (0.69-1.29) |
| Akko*1998-2000 | 1.18 | (0.90-1.56) |
| Haifa*1998-2000 | 1.03 | (0.83-1.27) |
| Hadera*1998-2000 | 1.01 | (0.73-1.41) |
| Hasharon*1998-2000 | 0.91 | (0.68-1.22) |
| Petach-Tikvah*1998-2000 | 1.03 | (0.80-1.31) |
| Ramla*1998-2000 | 0.97 | (0.67-1.39) |
| Rehovot*1998-2000 | 1.11 | (0.86-1.44) |
| Tel-Aviv*1998-2000 | 0.99 | (0.81-1.20) |
| Ashkelon*1998-2000 | 1.02 | (0.79-1.31) |
| Beer-Sheva*1998-2000 | 1.04 | (0.80-1.36) |
| Jerusalem*2001-2003 (reference) | 1 |  |
| Tzfat*2001-2003 | 0.85 | (0.47-1.54) |
| Kineret*2001-2003 | 0.97 | (0.51-1.82) |
| Izrael*2001-2003 | 0.95 | (0.70-1.29) |
| Akko*2001-2003 | 0.97 | (0.74-1.29) |
| Haifa*2001-2003 | 0.97 | (0.78-1.20) |
| Hadera*2001-2003 | 0.86 | (0.62-1.19) |
| Hasharon*2001-2003 | 0.77 | (0.57-1.03) |
| Petach-Tikvah*2001-2003 | 0.91 | (0.71-1.17) |
| Ramla*2001-2003 | 1.03 | (0.73-1.46) |
| Rehovot*2001-2003 | 0.95 | (0.73-1.24) |
| Tel-Aviv*2001-2003 | 0.87 | (0.72-1.06) |
| Ashkelon*2001-2003 | 0.90 | (0.70-1.17) |
| Beer-Sheva*2001-2003 | 0.81 | (0.62-1.07) |
| Jerusalem*2004-2006 (reference) | 1 |  |
| Tzfat*2004-2006 | 1.20 | (0.66-2.18) |
| Kineret*2004-2006 | 1.02 | (0.55-1.87) |
| Izrael*2004-2006 | 1.04 | (0.76-1.41) |
| Akko*2004-2006 | 1.17 | (0.88-1.54) |
| Haifa*2004-2006 | 1.16 | (0.94-1.44) |
| Hadera*2004-2006 | 1.03 | (0.73-1.43) |
| Hasharon*2004-2006 | 1.23 | (0.92-1.64) |
| Petach-Tikvah*2004-2006 | 1.00 | (0.78-1.29) |
| Ramla*2004-2006 | 1.11 | (0.77-1.59) |
| Rehovot*2004-2006 | 1.06 | (0.81-1.37) |
| Tel-Aviv*2004-2006 | 1 | (0.82-1.21) |
| Ashkelon*2004-2006 | 1.12 | (0.86-1.45) |
| Beer-Sheva*2004-2006 | 1.05 | (0.80-1.38) |
| Jerusalem*1998-2000 (reference) | 1.00 |  |
| Tzfat*2007-2009 | 1.69 | (0.94-3.04) |
| Kineret*2007-2009 | 1.33 | (0.73-2.43) |
| Izrael*2007-2009 | 1.20 | (0.87-1.66) |
| Akko*2007-2009 | 1.16 | (0.87-1.56) |
| Haifa*2007-2009 | 1.19 | (0.95-1.51) |
| Hadera*2007-2009 | 1.31 | (0.93-1.85) |
| Hasharon*2007-2009 | 1.00 | (0.73-1.37) |
| Petach-Tikvah*2007-2009 | 1.25 | (0.96-1.62) |
| Ramla*2007-2009 | 1.47* | (1.00-2.15) |
| Rehovot*2007-2009 | 1.09 | (0.83-1.45) |
| Tel-Aviv*2007-2009 | 1.26* | (1.02-1.56) |
| Ashkelon*2007-2009 | 1.08 | (0.82-1.43) |
| Beer-Sheva*2007-2009 | 1.42* | (1.07-1.88) |

^≠^Adjusted for age, sex, ethnicity districts and districts*year.

*p < 0.05, **p < 0.01, ***p < 0.001.

**Table S6: Adjusted^≠^ all-cause mortality hazards ratios**

| **Study variable** | **HR** | **(95% CI )** |
| --- | --- | --- |
|  |  |  |
| **Sex (male)** | 1.11*** | (1.08-1.15) |
| **Ethnicity (Jew)** | 0.72*** | (0.67-0.76) |
| **Age (per year)** | 1.05*** | (1.05-1.05) |
|  |  |  |
| Jerusalem * 1995-1997 (reference) | 1 |  |
| Jerusalem * 1998-2000 | 1.01 | (0.85-1.21) |
| Jerusalem * 2001-2003 | 1.01 | (0.85-1.21) |
| Jerusalem* 2004-2006 | 0.86 | (0.72-1.02) |
| Jerusalem * 2007-2009 | 0.73** | (0.60-0.88) |
| Tzfat * 1995-1997 | 0.93 | (0.60-1.44) |
| Tzfat * 1998-2000 | 0.96 | (0.64-1.43) |
| Tzfat * 2001-2003 | 0.80 | (0.54-1.20) |
| Tzfat * 2004-2006 | 0.96 | (0.64-1.44) |
| Tzfat * 2007-2009 | 1.14 | (0.77-1.69) |
| Kineret * 1995-1997 | 1.10 | (0.69-1.76) |
| Kineret * 1998-2000 | 1.33 | (0.96-1.85) |
| Kineret * 2001-2003 | 1.08 | (0.70-1.65) |
| Kineret * 2004-2006 | 0.96 | (0.65-1.42) |
| Kineret * 2007-2009 | 1.07 | (0.73-1.55) |
| Izrae l* 1995-1997 | 1.12 | (0.90-1.34) |
| Izrael * 1998-2000 | 1.07 | (0.86-1.33) |
| Izrael * 2001-2003 | 1.08 | (0.87-1.34) |
| Izrael * 2004-2006 | 0.99 | (0.79-1.24) |
| Izrael * 2007-2009 | 0.98 | (0.78-1.23) |
| Akko * 1995-1997 | 1.05 | (0.85-1.29) |
| Akko * 1998-2000 | 1.26* | (1.04-1.52) |
| Akko * 2001-2003 | 1.03 | (0.85-1.25) |
| Akko * 2004-2006 | 1.05 | (0.86-1.27) |
| Akko * 2007-2009 | 0.88 | (0.72-1.08) |
| Haifa * 1995-1997 | 0.94 | (0.80-1.10) |
| Haifa * 1998-2000 | 0.98 | (0.84-1.14) |
| Haifa * 2001-2003 | 0.92 | (0.79-1.09) |
| Haifa * 2004-2006 | 0.93 | (0.80-1.09) |
| Haifa * 2007-2009 | 0.81* | (0.69-0.96) |
| Hadera * 1995-1997 | 1.13 | (0.89-1.44) |
| Hadera * 1998-2000 | 1.16 | (0.92-1.47) |
| Hadera * 2001-2003 | 0.99 | (0.79-1.24) |
| Hadera * 2004-2006 | 0.99 | (0.78-1.26) |
| Hadera * 2007-2009 | 1.08 | (0.85-1.37) |
| Hasharon * 1995-1997 | 1.08 | (0.88-1.34) |
| Hasharon * 1998-2000 | 1.00 | (0.81-1.23) |
| Hasharon * 2001-2003 | 0.84 | (0.68-1.04) |
| Hasharon * 2004-2006 | 1.14 | (0.92-1.34) |
| Hasharon * 2007-2009 | 0.79* | (0.62-0.99) |
| Petach-Tikvah * 1995-1997 | 0.98 | (0.82-1.17) |
| Petach-Tikvah * 1998-2000 | 1.02 | (0.85-1.21) |
| Petach-Tikvah * 2001-2003 | 0.90 | (0.76-1.08) |
| Petach-Tikvah * 2004-2006 | 0.84 | (0.70-1.01) |
| Petach-Tikvah * 2007-2009 | 0.88 | (0.73-1.07) |
| Ramla * 1995-1997 | 1.14 | (0.88-1.47) |
| Ramla * 1998-2000 | 1.11 | (0.86-1.44) |
| Ramla * 2001-2003 | 1.19 | (0.94-1.50) |
| Ramla * 2004-2006 | 1.08 | (0.84-1.39) |
| Ramla * 2007-2009 | 1.21 | (0.92-1.59) |
| Rehovot * 1995-1997 | 0.96 | (0.80-1.16) |
| Rehovot * 1998-2000 | 1.08 | (0.90-1.29) |
| Rehovot * 2001-2003 | 0.92 | (0.76-1.12) |
| Rehovot * 2004-2006 | 0.87 | (0.72-1.05) |
| Rehovot * 2007-2009 | 0.76** | (0.63-0.93) |
| Tel-Aviv * 1995-1997 | 1.01 | (0.88-1.17) |
| Tel-Aviv * 1998-2000 | 1.01 | (0.88-1.17) |
| Tel-Aviv * 2001-2003 | 0.89 | (0.77-1.03) |
| Tel-Aviv * 2004-2006 | 0.86* | (0.75-0.99) |
| Tel-Aviv * 2007-2009 | 0.93 | (0.80-1.08) |
| Ashkelon * 1995-1997 | 1.21* | (1.00-1.45) |
| Ashkelon * 1998-2000 | 1.24* | (1.04-1.49) |
| Ashkelon * 2001-2003 | 1.11 | (0.92-1.33) |
| Ashkelon * 2004-2006 | 1.16 | (0.96-1.40) |
| Ashkelon * 2007-2009 | 0.95 | (0.78-1.16) |
| Beer-Sheva * 1995-1997 | 1.19 | (0.98-1.45) |
| Beer-Sheva * 1998-2000 | 1.26* | (1.04-1.52) |
| Beer-Sheva * 2001-2003 | 0.98 | (0.81-1.20) |
| Beer-Sheva * 2004-2006 | 1.07 | (0.88-1.31) |
| Beer-Sheva * 2007-2009 | 1.23* | (1.01-1.49) |

^≠^Adjusted for age, sex, ethnicity districts and districts*year.

*p < 0.05, **p < 0.01, ***p < 0.001.
